# Effect of Cycling Exercise Resisting Electrically Stimulated Antagonist Muscle Contractions in Healthy Males

**DOI:** 10.3390/metabo13050604

**Published:** 2023-04-28

**Authors:** Masayuki Omoto, Yuya Tsukada, Ryuki Hashida, Hiroo Matsuse, Hiroshi Tajima, Sohei Iwanaga, Yoshio Takano, Takeshi Nago, Yoshihiko Tagawa, Naoto Shiba

**Affiliations:** 1 Department of Orthopedics, Kurume University School of Medicine, Kurume 830-0011, Fukuoka, Japan; 2 Rehabilitation Center, Kurume University, Kurume 830-0011, Fukuoka, Japan; 3 Department of Physical Therapy, School of Health Sciences, International University of Health and Welfare, Okawa 831-8501, Fukuoka, Japan

**Keywords:** a cycle ergometer, metabolic cost, oxygen uptake, muscle strength, lactate, electrical stimulation

## Abstract

A hybrid training system (HTS) combining antagonist muscle electrical stimulation and voluntary muscle contraction has been developed using eccentric antagonist muscle contractions with electrical stimulation as resistance to voluntary muscle contractions. We devised an exercise method using HTS combined with a cycle ergometer (HCE). The purpose of this study was to compare the muscle strength, muscle volume, aerobic functions and lactate metabolism of HCE and a volitional cycle ergometer (VCE). A total of 14 male participants performed exercise on a bicycle ergometer for 30 min per session, 3 times per week for 6 weeks. We divided 14 participants into an HCE group (7 participants) and a VCE group (7 participants). The workload was set at 40% of each participant’s peak oxygen uptake ($\overset{.}{V}$O_2_peak). Electrodes were placed over each motor point on the quadriceps and hamstrings. The $\overset{.}{V}$O_2_peak and anaerobic threshold significantly increased before and after training when using HCE rather than VCE. The HCE group had significantly increased extension and flexion muscle strength at 180 degrees/s in post-training measurements over pre-training measurements. Knee flexion muscle strength at 180 degrees/s tended to increase in the HCE group compared to the VCE group. The quadricep muscle cross-sectional area was significantly increased in the HCE group compared to the VCE group. Additionally, the HCE group had significantly decreased maximal lactate, measured every 5 min during exercise at the end of study, between pre and post-training. Thus, HCE may be a more effective training method for muscle strength, muscle mass and aerobic functions at 40% of each participant’s $\overset{.}{V}$O_2_peak than conventional cycling exercise. HCE could be applied not only as aerobic exercise but also as resistance training.

## 1. Introduction

**Figure 1:**
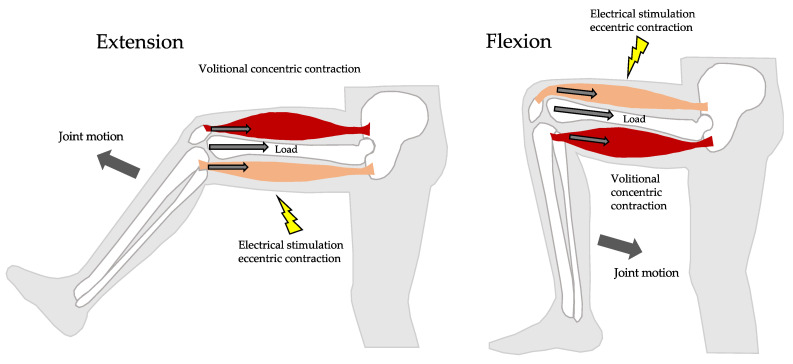
A hybrid training system (HTS) creates resistance to the motion of a voluntary contracting agonist muscle by means of the force generated by its electrically stimulated antagonist. It has both electrical stimulation and voluntary movement effects.

Various methods of simultaneous resistance and aerobic exercise have been developed, including HIIT (high-intensity interval training) [1], jumping squats, and circuit training [2]. Jackson J. Fyfe et al. reported that despite the potentially additive benefits of combining resistance and endurance exercise, there is considerable evidence that concurrent training compromises the development of muscle mass, strength, and power compared with undertaking resistance exercise alone [3]. Cycling exercises are commonly known as aerobic exercises and are effective as strength training at high loads [4]. On the other hand, neuromuscular electrical stimulation (NMES) is known to be able to increase muscle mass [5,6], strength [6,7] and physical performance [8]. The combined application of electrical stimulation and volitional contractions (VC) is said to be more effective than electrical stimulation or VC alone [9]. A hybrid training system (HTS) that creates resistance to the motion of a voluntary contracting agonist muscle by means of the force generated by its electrically stimulated antagonist has been developed (Figure 1) [10]. HTS has been shown to improve both muscle hypertrophy and strength while exercising joint flexion and extension, not only in healthy young participants [11] but also in elderly participants [12]. HTS can also place a resistance load on the quadriceps and hamstrings during cycling exercise. On the other hand, electrical muscle stimulation can also add aerobic loading. Moritani et al. reported that eleven healthy male participants performed moderate intensity pedaling exercise at a constant workload (80% of ventilatory threshold) for 20 min, while NMES was applied to thigh muscles from the 5 to 10 min mark and from the 15 to 20 min mark during the exercise [13]. Oxygen uptake ($\overset{.}{V}$O_2_), heart rate, and the respiratory gas exchange ratio were significantly higher during the exercise periods with NMES than during the unassisted workload. These changes were accompanied by an elevated blood lactate concentration, suggesting the existence of additional fast-twitch motor unit recruitment and activation of glucose metabolic circuits during the exercise with NMES. So Young Lee et al. reported that sixteen subacute stroke patents were randomly assigned to a functional electrical stimulation (FES) group (*n* = 8) or a control group (*n* = 8) [14]. All patients underwent assistive ergometer training for 30 min (5 times per week for 4 weeks). The electrical stimulation group received FES on the paretic lower limb muscles during assistive ergometer training. At 4 weeks after treatment, the FES-assisted ergometer training group showed significant improvements in the six-minute walk test (*p* = 0.01), the Berg Balance Scale (*p* = 0.01), the Korean version of Modified Barthel Index (*p* = 0.01), $\overset{.}{V}$O_2_peak (*p* = 0.02), metabolic equivalent (*p* = 0.02), and estimated anaerobic threshold (*p* = 0.02). The study showed that FES-assisted ergometer training improved aerobic capacity. We reported in a previous study that the combination of cycling and HTS increased oxygen uptake about 2.1 ± 0.17 mL/kg/min from aerobic cycle ergometer exercise at moderate intensity [15]. Furthermore, similar to conventional aerobic ergometer exercise, oxygen uptake and heart rate during HCE had a linear relationship to workload. It is generally known that electrical stimulation acts on fast-twitch muscles and produces lactate [16]. In our previous study, lactate increased immediately after 30 min of HCE, but not significantly compared to cycling alone. We noted that this was due to the lactate shuttle, in which HTS produces lactate in fast-twitch muscles and utilizes it as an energy source in slow-twitch muscles [17]. Therefore, we suppose that the combination of cycling and HTS is useful as not only an aerobic exercise but also resistance training. We conducted this study as a step to developing an efficient exercise method that simultaneously combines resistance exercise and aerobic exercise at low loads. We examined the effects of 6 weeks of HTS with cycling on lactate metabolic pathways, muscle strengthening, and aerobic exercise.

## 2. Participants and Methods

### 2.1. Participants

**Figure 2:**
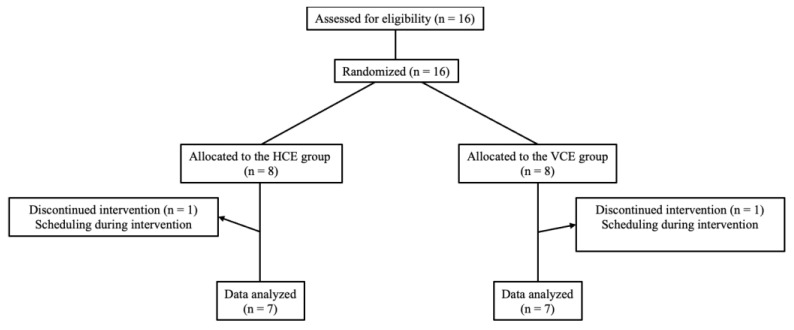
Diagram of participants’ flow throughout the study.

Sixteen healthy young men [mean ± standard deviation (SD): age 20.7 ± 0.5 years; height 169.7 ± 7.9 cm; body mass 61.2 ± 8.5 Kg] agreed to participate. All procedures were fully explained to the participants, who gave their written informed consent to participate. They were randomly divided by a blinded assessor using a computer into two groups: HTS combined with cycle ergometer (HCE) and a volitional cycle ergometer (VCE) group. We excluded two participants for physical fitness reasons. The HCE group, who trained with HTS during ergometer exercise, consisted of 7 participants, and the VCE group consisted of 7 participants who trained by ergometer exercise only (Figure 2). After giving written consent, participants were examined by an orthopedic specialist who was not involved in this study. The exclusion criteria for the training intervention were cases of acute orthopedic problems in the upper or lower extremities, congenital heart disease, pulmonary disease or pituitary disease. The inclusion criterion was that participants were nonsmokers. Exercise was prohibited during the intervention period and for two weeks before and after the intervention. The participants were instructed to avoid excessive alcohol consumption. They had passed an examination for normal activity of daily life, strength, sensation, and range of motion according to the criteria of the Japanese Orthopedic Association. All participants underwent medical, musculoskeletal examinations and cardiopulmonary exercise tests (CPX) conducted by a physician and/or physical therapist. The study was conducted in adherence with the standards of the World Medical Association Declaration of Helsinki: Ethical Principles for Medical Research Involving Human Subjects (2013 version). The Ethics Committee of Kurume University approved the clinical design of this study protocol (approval ID: 12059).

### 2.2. Training Protocol

The ergometer exercise was conducted for 30 min per session, 3 times a week for 6 weeks (a total of 18 sessions). Each session was separated by an interval of at least 48 h. All the sessions were conducted at the University Laboratory. The participants were instructed to carry on their ordinary daily lives. They were prohibited from participating in new activities for the purpose of physical fitness and/or physical strength improvement. All participants were measured for height and body mass and performed the ramp exercise test to determine their $\overset{.}{V}$O_2_peak and anaerobic threshold. The ramp exercise test protocol was performed on an electronically braked cycle ergometer (75XL III, Konami, Tokyo, Japan) until participants were not able to maintain their cadence above 60 revolutions/min. To alleviate buttock pain in participants who were not accustomed to cycling, we tilted the saddle angle backward by 10 degrees. The knee joint range of motion was set at a nearly 90° arc that extended from 20° flexion to 110° by adjusting the height of the saddle.

### 2.3. VCE Group Protocol

The cycling exercise load was set at 40% of each participant’s $\overset{.}{V}$O_2_peak (the workload in VCE group; 91.43 ± 6.90 watts). Every training session began and ended with 5 min of stretching guided by the assistant. After a 2 min warm up at 30 watts, the participants began the actual exercise protocol [18]. Pedaling cadence was kept constant at 60–80 rev/min with the aid of a pedal frequency meter. During the exercise, an assistant was always present to provide guidance and monitoring in order to ensure that the exercise was performed safely and properly.

### 2.4. HCE Group Protocol

HTS was performed simultaneously with a volitional cycle ergometer, with the participant’s hamstrings electrically stimulated as he volitionally extended his knee, and his quadriceps electrically stimulated as he volitionally flexed his knee, in order to provide motion resistance. The pedaling cadence was kept at 60–80 rev/min. During HCE, both lower extremities were stimulated corresponding to the bending motions of the knee using HTS. Every training session began and ended with 5 min of stretching guided by the assistant. After a 2 min warm up at 30 watts, the participants began the actual exercise protocol [18]. Each session was separated by an interval of at 48 h. All sessions were conducted at the University laboratory. Pedaling cadence was kept constant at 60–80 rev/min with the aid of a pedal frequency meter. Participants were prohibited from participating in new activities for the purpose of resistance movement and physical strength improvement. During the exercise, an assistant was always present to provide guidance and monitoring in order to ensure that the exercise was performed safely and properly. 2 min HTS and 1 min rest intervals were repeated during the ergometer exercise for 30 min. We reported in a previous study that the oxygen uptake during HCE has a linear relationship with workload just as in a conventional ergometer, and that exercise intensity could be adjusted using heart rate [15]. The oxygen uptake during HCE was significantly higher than during VCE at an average of 21.2%. Based on those data, we calculated the workload (target heart rate) using a linear regression equation and adjusted it during HCE in order to attain the same oxygen uptake as that attained during VCE. Therefore, the ratio of oxygen uptake to $\overset{.}{V}$O_2_peak in both the HCE and VCE group was the same, at 40%. The mean heart rate of the HCE group was not significantly different from that of the VCE group.

### 2.5. Electrical Stimulation Protocol

**Figure 3:**
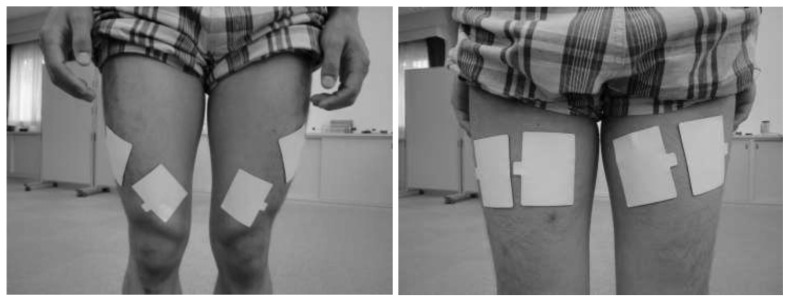
Electrodes were placed over each motor point on the quadriceps and hamstrings.

The electrical stimulation device can be described as a previously designed waveform generator capable of delivering stimulating signals with unique frequencies and waveforms to as many as four pairs of electrodes and a joint motion sensor (Mutoh Engineering Inc., Tokyo, Japan). The device triggers stimulation of the antagonist once it senses the initiation of an agonist’s volitional contraction. We searched for motor points in the quadriceps and hamstring muscles prior to intervention by applying electrodes to the presumed motor points and checking where muscle contractions were seen with as little current as possible. Pairs of 3 × 6 cm low-impedance gel-coated carbon electrodes (Sekisui Chemical Co., LTD, Tokyo, Japan) were placed over each motor point on the quadriceps and the hamstrings, and a detector was also attached (Figure 3). The position of the left pedal was the highest at 0 degrees. The participant sat on the cycle ergometer (KONAMI 75XL III; Konami Sports Co., LTD, Tokyo, Japan) with his hamstrings electrically stimulated as he volitionally extended his knee, and his quadriceps electrically stimulated as he volitionally flexed his knee. The timing of the electrical stimulation was controlled by a joint motion sensor attached to knee.

### 2.6. Evaluations

All the evaluations were performed by a blinded assessor one week before and after the training, respectively. The maximal isokinetic torque of knee extension was measured on the non-dominant lower extremities. All the evaluations were performed by one physical therapist and two assistants. Maximal volitional isokinetic knee extension/flexion torques were measured at angular velocities of 60°/s and 180°/s with the BIODEX SYSTEM 3 PRO (Biodex Medical Systems Inc., Shirley, NY, USA) [19]. During the strength measurements, the participant was seated on the Biodex in an upright position. Velcro belts were applied to fix the trunk and thigh in position. The seat was adjusted to the same position at each evaluation. Each session began by establishing that the participant could move his lower extremity comfortably throughout the full 20–100° arc of the exercise range. He then performed three practice contractions in the direction and at the speed to be tested. A measurement session consisted of three sets separated by 3 min after the practice; the three measurements from the non-dominant lower extremities were pooled, and the mean was adjusted according to each body mass (kg) used for statistical analysis. We measured a cross-section of quadriceps and hamstrings before and after the training program by means of magnetic resonance imaging (MRT-50A Filexeret; Toshiba Medical System Co., Tochigi, Japan). A marker was placed halfway below the line connecting the long anterior iliac crest and the upper edge of the patella. The scan files of pre-training were compared with those of post-training. The same scan slice of the muscle locations in the CSAs was selected concerning the entire quadriceps and hamstrings femoris. These files were imported to a Windows XP computer to be analyzed. The imported muscle CSAs were all analyzed by a blinded assessor using NIH Image software (Image J, version 1.451; NIH, Bethesda, MD, USA). The muscle force per unit area was defined as the muscle force per muscle cross-sectional area.

### 2.7. Blood Lactate Concentration

Maximal blood lactate concentration values were measured every 5 min at the beginning and during the last cycling exercise. We measured finger-prick blood using the Lactate Pro blood lactate analyzer (ARKRAY Factory, lnc., Shiga, Japan) [20].

### 2.8. Statical Analysis

All the statistical analyses were performed by JMP Version 13.0 statistical software (SAS Institute Inc., Cary, NC, USA), and values of *p* < 0.05 were considered to be statistically significant in all cases. A Shapiro–Wilk test was used to check normal distribution. Average and standard deviations were calculated for all measurements. The Wilcoxon rank sum test and a two-sample *t*-test were used to test for differences in baseline characteristics between the groups. The Wilcoxon signed-rank test and paired *t*-test were used to test for changes from baseline in each training group before and after the six-week training period. To compare changes from baseline between the two training groups, the data analyzed were tested with a Wilcoxon rank sum test and two-sample *t*-test. Moreover, we calculated the effect size of Cohen’s d to determine the strength of association between interventions.

## 3. Results

### 3.1. Baseline Characteristics

Table 1 shows the characteristics of the two groups. Knee extension and flexion muscle strength at 180 degrees angular velocity was significantly greater in HCE, but other baseline clinical features were similar.

### 3.2. Muscle Strength

Knee extension and flexion muscle strength at 60 degrees angular velocity did not increase significantly in the VCE group. However, the HCE group showed a significant increase in flexion muscle strength at 60 degrees angular velocity (knee flexor strength in the HCE group was from 78.74 ± 13.71 [N·m·kg^−1^] to 94.30 ± 15.63 [N·m·kg^−1^], *p* = 0.0373) (Table 2). The HCE group showed a significant increase in extension and flexion muscle strength at 180 degrees angular velocity, but there was no significant difference in the VCE group (knee extensor strength in the HCE group was from 76.49 ± 22.23 [N·m·kg^−1^] to 95.41 ± 18.33 [N·m·kg^−1^], *p*= 0.0491, and knee flexor strength in the HCE group was from 44.97 ± 10.63 [N·m·kg^−1^] to 67.54 ± 20.06 [N·m·kg^−1^], *p* = 0.0042) (Table 2). Comparing the differences in average values between before and after training in the two groups, there was a tendency for knee flexor strength at 180 degrees angular velocity to increase in the HCE group (95% confidence interval [CI] of knee flexor: −0.98, 33.78; *p* = 0.06) (Table 3). The effect size *d* of knee extension muscle strength at 180°/s in the HCE group was 0.85. The effect size *d* of knee flexion muscle strength at 180°/s in the HCE group was 2.13.

### 3.3. Muscle Mass

In the HCE group, the quadricep and hamstring muscle cross-sectional areas increased significantly after training (the cross-sectional area of the quadricep muscles of HCE group increased from 6866.24 ± 613.32 [mm^2^] pre-training to 7587.55 ± 812.02 [mm^2^] post-training (*p* = 0.0002)) (the cross-sectional area of hamstring muscles of the HCE group increased from 6310.02 ± 1291.11 [mm^2^] pre-training to 6670.20 ± 1509.56 [mm^2^] post-training (*p* = 0.0313)) (Table 2). The effect sizes *d* in the cross-sectional areas of the quadricep and hamstring muscles were 1.18 and 0.28 in the HCE group. In the VCE group, there was no significant increase in the quadricep muscle cross-sectional area. However, the hamstring muscle’s cross-sectional area was significantly increased (the cross-sectional area of hamstring muscle of the VCE group increased from 7169.85 ± 568.80 [mm^2^] pre-training to 7459.84 ± 499.84 [mm^2^] post-training (*p* = 0.0313)) (Table 2). The effect size *d* in the cross-sectional area of the hamstring muscle was 0.51 in the VCE group. In addition, the change in the quadricep muscle cross-sectional area was significantly greater in the HCE group than in the VCE group (95% CI: 217.88, 823.15; *p* = 0.0028) (Table 3). The change in the hamstring muscle’s cross-sectional area was not statistically significant (95% CI: −284.33, 424.71; *p* = 0.6738) (Table 3). The muscle force per unit area was defined as the muscle force per muscle cross-sectional area. The flexion muscle strength per unit area at 180 degrees angular velocity was significantly increased in the HCE group (*p* = 0.0476). However, there was not a statistically significant difference in the VCE group (*p* = 0.6875). The change in flexion muscle strength per unit area at 180 degrees angular velocity was statistically significant in the HCE group compared to the VCE group (95% CI: 0.000394, 0.005163; *p* = 0.0476). The effect sizes *d* in the HCE group and the VCE group were 1.49 and 0.19.

### 3.4. Peak Oxygen Uptake ($\overset{.}{V}$O_2_peak)

There was no change in $\overset{.}{V}$O_2_peak in the VCE group, but there was a significant increase in the HCE group. (VCE group: from 39.27 ± 3.51 [mL·Kg^−1^·min^−1^] pre-training to 39.17 ± 2.60 [mL·Kg^−1^·min^−1^] post-training (*p* = 0.9276), and HCE group: from 40.50 ± 4.10 [mL·Kg^−1^·min^−1^] pre-training to 44.17 ± 5.50 [mL·Kg^−1^·min^−1^] post-training (*p* = 0.0247)) (Table 2). The effect sizes *d* of $\overset{.}{V}$O_2_peak in the VCE group and the HCE group were −0.3 and 0.9. In addition, change in $\overset{.}{V}$O_2_peak in the HCE group was significantly greater than in the VCE group (95% CI: 0.24, 7.31; *p* = 0.0385) (Table 3).

### 3.5. Anaerobic Threshold

There was no change in the anaerobic thresholds in the VCE group, but there was a significant increase in the HCE group. (VCE group: from 18.93 ± 1.84 [mL·Kg^−1^·min^−1^] pre-training to 19.43 ± 2.23 [mL·Kg^−1^·min^−1^] post-training (*p* = 0.4628) and HCE group: from 18.40 ± 1.60 [mL·Kg^−1^·min^−1^] pre-training to 22.99 ± 2.36 [mL·Kg^−1^·min^−1^] post-training (*p* = 0.0028) (Table 2). The effect sizes *d* of the anaerobic thresholds in the VCE group and the HCE group were 0.27 and 2.9. In addition, the change in the anaerobic thresholds in the HCE group was significantly greater than in the VCE group (95% CI: 1.60, 6.57; *p* = 0.0127) (Table 3).

### 3.6. Peak Blood Lactate

There was no significant difference in resting blood lactate in both groups before and after training. There was no change in peak blood lactate in the VCE group, but in the HCE group, peak blood lactate was significantly reduced from 8.86 ± 3.18 mmol/L at pre-training to 5.29 ± 1.48 [mmol/L] at post-training (*p* = 0.0156) (Table 2). The effect sizes *d* of peak blood lactate in the VCE group and the HCE group were −0.57 and −1.12. However, there was no significant difference in change of peak blood lactate between the HCE and the VCE groups (95% CI: −5.32, 2.14; *p* = 0.3079) (Table 3).

## 4. Discussion

We conducted this study as a step to developing an efficient exercise method that simultaneously combines resistance exercise and aerobic exercise at low loads. We examined the effects of 6 weeks of HTS with cycling as aerobic exercise on muscle strengthening and the lactate metabolic pathways. The $\overset{.}{V}$O_2_peak and the anaerobic thresholds were significantly increased in the HCE group compared to the VCE group. Knee flexion muscle strength at 180 degrees/s tended to increase in the HCE group. The quadricep muscle’s cross-sectional area was significantly increased in the HCE group compared to the VCE group. Thus, HCE may be a more effective training method at 40% of each participant’s $\overset{.}{V}$O_2_peak for muscle strength, muscle mass and aerobic functions than conventional cycling exercise.

There is evidence that HTS is effective as a device for increasing lower limb muscle strength. Takano et al. investigated the effect of HTS on knee extension muscle strength in elderly individuals, giving due consideration to their safety [12]. Twenty elderly individuals who met exclusion criteria such as brain and heart diseases were randomly assigned to two groups: an HTS group, and a large weight training machine (WT) group. Both groups performed training twice a week for 12 weeks, consisting of 10 repetitions of knee flexion and extension movements for 3 s each. After training, knee extension torque increased significantly in both groups (39% in the HTS group and 42% in the WT group), and the overall area of the quadriceps femoris muscle increased significantly in both groups (9% in the HTS group and 14% in the WT group). These results demonstrated that the portable and small HTS was effective in increasing muscle strength and mass, similar to the large training machine. In this study, it was found that HTS could be applied to a bicycle ergometer and increase muscle strength and mass, suggesting its potential for practical use.

Sylvain Dorel et al. reported that EMG measurements with an average 200 ± 12 W ergometer showed that the biceps femoris, semimembranosus, vastus medialis, and vastus lateralis muscles were muscularly active during hip extension and knee extension, while little muscle activity was observed during hip flexion and knee flexion, except for the rectus femoris muscle [21]. In the present study, HCE significantly increased extension and flexion muscle strength at 180 degrees/s between pre- and post-training. Knee flexion muscle strength at 180 degrees/s tended to increase in the HCE group compared to the VCE group. This is thought to be because HTS stimulates eccentric electrical contraction to the biceps femoris muscle during hip and knee extension, and eccentric electrical contraction to the quadriceps femoris muscle during hip and knee flexion, which allows resistance exercise to be continuously performed, thereby allowing efficient muscle strengthening training.

We have confirmed in previous studies that HCE exercise induces the secretion of growth hormone, which may contribute to the induction of skeletal muscle hypertrophy. Generally, muscle hypertrophy takes six weeks. Tibor Hortoba’gyi et al. reported that electrically stimulated strength training performed for 6 weeks at clinical doses and parameters produces changes in muscle metabolism and promotes minor isoform shifts, but the increase in maximal voluntary contraction is not the result of overt muscle hypertrophy; instead, itis mediated by changes in several elements of the nervous system [22]. In our study, the muscle cross-sectional area for HCE was significantly increased in the quadriceps and the hamstrings between pre- and post-training. However, the change in the hamstring muscle’s cross-sectional area was not statistically significant between the HCE and VCE groups. The muscle strength per unit area was increased in the hamstrings significantly. The increase in muscle strength per unit area in the present study was thought to be due to neural adaptation.

In a previous study, we reported that adding HTS to cycling exercise increased $\overset{.}{V}$O_2_ by approximately 20% [15]. Based on those data, we calculated the workload (target heart rate) using a linear regression equation and adjusted it during HCE to attain the same oxygen uptake as during VCE. In this study, despite HCE having the same 40% $\overset{.}{V}$O_2_ as VCE, both the $\overset{.}{V}$O_2_peak and anaerobic threshold values were significantly increased in HCE. This is likely due to the following mechanism, proposed by L. B. Gladden, in which lactate generated in fast-twitch fibers is oxidized in cardiac and slow-twitch fibers, thereby becoming a source of energy [23]. The effect size for lactate level in this study was large in the HCE group (d = −1.12) and small in the VCE group (d = −0.57), indicating that lactate was more efficiently utilized as an energy source in the HTS group. This may be because lactate produced by forced muscle contractions in antagonistic muscles during NMES was utilized in slow-twitch muscles that contract voluntarily in agonistic muscles, leading to the activation of mitochondrial function in slow-twitch fibers and an improvement in aerobic capacity. It has also been reported that NMES improves mitochondrial function in skeletal muscles and increases muscle oxygen uptake [24,25]. In summary, it is thought that the improvement of mitochondrial function through the lactate shuttle mechanism contributed to the improvement of aerobic function in the HTS group. Moreover, Craig, D.M. et al. stated that following endurance training, the shift in whole-body substrate oxidation toward greater lipid oxidation and reduced glycolysis allows for a greater absolute exercise intensity to be supported predominantly by aerobic energy production [26]. They stated that lactate accumulation in both the blood and muscles decreases as a result. In our own research, lactate significantly decreased after 6 weeks of HCE training. The effect size was stronger in the HCE group than in the VCE group, but the lack of significant differences in the lactate peak between the groups is likely due to the small number of participants. If the number of participants increases, significant differences in lactate peak between the groups are expected to emerge.

In our study, aerobic and resistance exercise effects were obtained in HCE, although the training was performed at a low load, that is, below the anaerobic threshold. This may be applicable to patients with frailty or underlying conditions who have difficulty with high load pedaling.

There are a few potential limitations of this study. Although we found significant differences between HCE and VCE, the limited number of participants should be considered. HCE results seemed to differ according to the height and weight of the participant compared to VCE; however, this difference was not statistically significant. There was a difference between HCE and VCE in knee extension muscle strength at 180 degrees/s at baseline. More analysis of results is needed. Our study only included young male adults and our results cannot be extended to other populations. Additionally, we have not trialed other exercise intensities. Our results were not compared with electrical stimulation alone without cycling exercise. As the results may differ depending on differences in conditions such as saddle position, it is necessary to investigate under various conditions. We have not collected tissue samples, so changes in muscle composition are unknown.

## 5. Conclusions

We adjusted the workload so that the ratio of oxygen uptake to $\overset{.}{V}$O_2_peak between HCE and VCE was the same, at 40%. Nevertheless, the $\overset{.}{V}$O_2_peak, anaerobic threshold, and quadricep muscle mass were increased in the HCE group compared to the VCE group. HCE could combine resistance exercise with aerobic exercise, even at a low load below the anaerobic threshold. HCE might facilitate both resistance exercise’s effect locally and aerobic exercise’s effect on the whole body.

## Figures and Tables

**Table 1:** Baseline characteristics of HCE and VCE groups.

	HCE			VCE			
	Mean		SD	Mean		SD	*p*
Height (cm)	168.57	±	4.04	171.43	±	11.49	0.55 ^#^
Body mass (Kg)	59.29	±	4.54	65.00	±	10.88	0.22 ^#^
Knee extension muscle strength at 60°/s (Nm/kg)	137.61	±	34.75	156.64	±	35.48	0.33 ^#^
Knee extension muscle strength at 180°/s (Nm/kg)	76.49	±	22.23	104.43	±	17.92	0.02 ^#^
Knee flexion muscle strength at 60°/s (Nm/kg)	78.74	±	13.71	92.53	±	17.22	0.12 ^#^
Knee flexion muscle strength at 180°/s (Nm/kg)	44.97	±	10.63	67.97	±	11.82	0.12 ^#^
Resting blood lactate (mmol/L)	2.24	±	0.81	2.14	±	1.12	0.85 ^#^
Peak blood lactate (mmol/L)	8.86	±	3.18	8.36	±	3.46	0.57 ^†^
Quadricep muscle cross-sectional area (mm^2^)	6866.24	±	613.32	8021.56	±	655.04	0.20 ^#^
Hamstring muscle cross-sectional area (mm^2^)	6310.02	±	1291.11	7169.85	±	568.80	0.57 ^†^
Peak oxygen uptake (mL/kg/min)	40.50	±	4.08	39.27	±	3.51	0.56 ^#^
Anaerobic thresholds (mL/kg/min)	18.40	±	1.58	18.93	±	1.84	0.58 ^#^

HCE, HTS combined with cycle ergometer; VCE, a volitional cycle ergometer. Data are expressed as means ± standard deviation. ^#^ *p*-values are for comparison of groups using a two-sample *t*-test. ^†^ *p*-values are for comparison of groups using a Wilcoxon rank sum test. Values of *p* < 0.05 were considered statistically significant in all cases.

**Table 2:** Changes after ergometer exercise for 6 weeks.

	HCE							VCE						
	Before			After			*p*	Before			After			*p*
Knee extension muscle strength at 60°/s (Nm/kg)	137.61	±	34.75	156.66	±	27.81	0.16 ^##^	156.64	±	35.48	173.21	±	32.02	0.18 ^##^
Knee extension muscle strength at 180°/s (Nm/kg)	76.49	±	22.23	95.41	±	18.33 *	0.049 ^##^	104.43	±	17.92	106.20	±	21.10	0.77 ^##^
Knee flexion muscle strength at 60°/s (Nm/kg)	78.74	±	13.71	94.30	±	15.63 *	0.04 ^##^	92.53	±	17.22	105.54	±	14.21	0.06 ^##^
Knee flexion muscle strength at 180°/s (Nm/kg)	44.97	±	10.63	67.54	±	20.06 *	0.01 ^##^	67.97	±	11.82	74.14	±	14.80	0.36 ^##^
Resting blood lactate (mmol/L)	2.24	±	0.81	1.97	±	0.88	0.60 ^##^	2.14	±	1.12	2.37	±	1.78	0.82 ^##^
Peak blood lactate (mmol/L)	8.86	±	3.18	5.29	±	1.48 *	0.02 ^††^	8.36	±	3.46	6.39	±	2.39	0.23 ^††^
Quadricep muscle cross-sectional area (mm^2^)	6866.24	±	613.32	7587.55	±	812.02 *	0.01 ^##^	8021.56	±	655.04	8222.35	±	743.07	0.10 ^##^
Hamstring muscle cross-sectional area (mm^2^)	6310.02	±	1291.11	6670.20	±	1509.56 *	0.03 ^††^	7169.85	±	568.80	7459.84	±	499.84 *	0.03 ^††^
Peak oxygen uptake (mL/kg/min)	40.50	±	4.10	44.17	±	5.50 *	0.02 ^##^	39.27	±	3.51	39.17	±	2.60	0.93 ^##^
Anaerobic thresholds (mL/kg/min)	18.40	±	1.60	22.99	±	2.36 *	0.01 ^##^	18.93	±	1.84	19.43	±	2.23	0.46 ^##^

HCE, HTS combined with cycle ergometer; VCE, a volitional cycle ergometer. Data are expressed as means ± standard deviation. ^##^ *p*-values are for comparison of before and after, using a paired *t*-test. ^††^ *p*-values are for comparison of before and after, using a Wilcoxon signed-rank test. Values of *p* < 0.05 were considered statistically significant in all cases. * *p* < 0.05.

**Table 3:** The difference between the average values of change before and after training in the two groups.

	Difference of Means (HCE-VCE)	95%CI	*p*
Knee extension muscle strength at 60°/s (Nm/kg)	2.47	−32.65, 37.59	0.70 ^α^
Knee extension muscle strength at 180°/s (Nm/kg)	17.16	−3.83, 38.15	0.14 ^α^
Knee flexion muscle strength at 60°/s (Nm/kg)	2.54	−14.94, 20.02	0.76 ^β^
Knee flexion muscle strength at 180°/s (Nm/kg)	16.40	−0.98, 33.78	0.06 ^β^
Peak blood lactate (mmol/L)	−1.59	−5.32, 2.14	0.31 ^β^
Quadricep muscle cross-sectional area (mm^2^)	520.51	217.88, 823.15	0.01 ^β^
Hamstring muscle cross-sectional area (mm^2^)	70.19	−284.33, 424.71	0.67 ^β^
Peak oxygen uptake (mL/kg/min)	3.77	0.24, 7.31	0.04 ^β^
Anaerobic thresholds (mL/kg/min)	4.09	1.60, 6.57	0.01 ^α^

HCE, HTS combined with cycle ergometer; VCE, a volitional cycle ergometer. ^α^ *p*-values are for comparison of groups using a Wilcoxon rank sum test. ^β^ *p*-values are for comparison of groups using a two-sample *t*-test.

## Data Availability

The data presented in this study are available on request from the corresponding author. The data are not publicly available due to privacy restrictions.
